# Oxidative stress and inflammation mediate the association between elevated oxidative balance scores and improved sleep quality: evidence from NHANES

**DOI:** 10.3389/fnut.2024.1469779

**Published:** 2024-10-18

**Authors:** Qu Zhang, Jing Yi, Yemei Wu

**Affiliations:** ^1^Department of Radiotherapy Center, Hubei Cancer Hospital, Tongji Medical College, Huazhong University of Science and Technology, Wuhan, China; ^2^School of Public Health, Tongji Medical College, Huazhong University of Science and Technology, Wuhan, China; ^3^Hubei Cancer Hospital, Tongji Medical College, Huazhong University of Science and Technology, Wuhan, China

**Keywords:** oxidative balance score, sleep quality, inflammation, mediation analysis, National Health and Nutrition Examination Survey

## Abstract

**Background:**

The association between oxidative stress, as measured by the Oxidative Balance Score (OBS), and sleep quality remains unclear. The primary objective of this investigation was to clarify this relationship and to explore the potential involvement of oxidative stress and inflammation.

**Methods:**

Data from 15,198 participants in the National Health and Nutrition Examination Survey 2007–2014 were analyzed. Sleep quality indicators, including sleep disorder, trouble, and duration, were assessed. The OBS, comprising information on 16 dietary nutrients and 4 lifestyle factors, was then calculated. Multivariable logistic and linear regression models were employed to investigate the correlation between OBS and sleep quality. Additionally, mediation analyses were conducted to evaluate the potential effects of oxidative stress and inflammation.

**Results:**

We demonstrated a correlation between an elevated OBS and reduced sleep disorders (OR, 0.72; 95% CI, 0.58–0.91; *p* = 0.0055), reduced sleep trouble (OR, 0.81; 95% CI, 0.69–0.96; *p* = 0.0174), and prolonged sleep duration (*β* 0.009; 95% CI, 0.0002–0.0160; *p* = 0.015) when comparing the highest and lowest tertiles. Dietary factors exhibited autonomous correlations with sleep duration, whereas lifestyle factors displayed independent associations with sleep trouble and sleep disorders. Moreover, the relationships between OBS and both sleep disorders and trouble were influenced by albumin, *γ*-glutamyl transferase, total bilirubin, and white blood cells, with combined mediation effects of 34.66 and 29.54%, respectively (both *p* < 0.001). Sensitivity analyses revealed a significant association between OBS and sleep disorder (*p* < 0.001).

**Conclusion:**

This study revealed a positive correlation between an elevated OBS and improved sleep quality, manifested by decreased sleep disorders, mitigated sleep trouble, and prolonged sleep duration. This is potentially mediated by oxidative stress and inflammation. Therefore, the study underscores the importance of adopting a diet rich in antioxidants and healthy lifestyle choices to address sleep-related concerns, providing a novel avenue for enhancing overall sleep quality.

## 1 Introduction

Sleep is a crucial biological activity for maintaining physical and mental health, influencing various physiological processes, including inflammatory responses (1, 2). Deterioration in sleep quality is closely linked to a range of chronic conditions such as depression, cognitive impairment, and cardiovascular diseases (3–5). Statistical data indicate that the prevalence of sleep disorders among American adults has reached 27.1% and continues to rise (6), highlighting sleep quality issues as a significant global public health concern (7). Research has shown that insufficient sleep can elevate the production of pro-inflammatory cytokines, such as TNF-*α*, IL-1, and IL-6, suggesting a correlation between sleep duration and inflammation-related diseases (8–12). Sleep disorders are also recognized as known risk factors for inflammatory diseases (e.g., rheumatoid arthritis and systemic lupus erythematosus) and mental disorders (e.g., major depressive disorder) (13–15). Therefore, improving sleep quality is not only associated with the restoration of individual physiological functions but may also play a vital role in the prevention and intervention of inflammation-related diseases.

In recent years, research has revealed that oxidative stress, characterized by an imbalance between antioxidants and pro-oxidants, leading to cellular damage and death, may be a crucial factor in determining sleep quality (16). Previous studies have associated increased levels of oxidative stress with sleep disorders and short sleep duration (17–20). Pro-oxidants induce oxidative stress by generating reactive oxygen species (ROS) or reducing the defense activity of antioxidant systems, while antioxidants may shift the balance towards a less oxidative state (21). Furthermore, supplementation of antioxidant nutrients such as vitamin C (22) and luteolin (23), and adoption of antioxidative lifestyle behaviors such as weight control (24) and physical exercise (25) have been shown to reduce oxidative stress levels and improve sleep quality.

Despite existing research exploring the relationship between oxidative stress and sleep quality, most studies have focused on isolated factors, failing to comprehensively consider the combined effects of diet and lifestyle on oxidative stress levels. To address this gap, the Oxidative Balance Score (OBS) has been proposed as a tool for a holistic assessment of individual oxidative stress, incorporating dietary and lifestyle factors. The OBS has been shown to be significantly associated with various chronic diseases, including type 2 diabetes, cardiovascular diseases, and colorectal cancer. However, there remains a lack of in-depth research regarding the specific mechanisms linking OBS to sleep quality, particularly the mediating roles of oxidative stress and inflammation.

Research indicates that a decline in sleep quality is closely associated with systemic inflammation, characterized by elevated levels of pro-inflammatory cytokines and oxidative stress biomarkers (26, 27). For instance, albumin (28) and bilirubin (29) possess strong antioxidant properties, while gamma-glutamyl transferase (GGT), linked to glutathione metabolism (30), serves as a key marker of oxidative stress. White blood cells (WBC) (31) act as indicators of inflammation, reflecting the body’s inflammatory status (32). High levels of OBS are negatively correlated with GGT levels and are associated with a reduction in other inflammatory markers such as WBC. These findings suggest that low OBS may adversely affect sleep quality by increasing oxidative stress and inflammatory responses.

To address the knowledge gap, we analyzed data from the National Health and Nutrition Examination Survey (NHANES) to explore the relationship between OBS and sleep quality, focusing on the mediating roles of oxidative stress and inflammation. This is the innovation of the article and what sets it apart from previous studies (16–20). We assessed sleep quality through self-reported duration, clinically diagnosed disorders, and subjective sleep disturbances, providing a comprehensive evaluation. By integrating dietary and lifestyle factors to calculate OBS, we found a positive association between higher OBS and improved sleep parameters. Furthermore, we examined biomarkers such as albumin, GGT, bilirubin, and WBC to explore the underlying biological mechanisms, offering new insights into how oxidative stress and inflammation mediate the OBS-sleep relationship. This study provides theoretical and practical contributions, supporting targeted interventions for improving sleep quality through diet and lifestyle modifications.

This study aims to investigate the association between OBS and sleep quality using data from the NHANES, with a particular focus on the mediating roles of oxidative stress and inflammation. By analyzing multiple dimensions of sleep-including sleep duration, objective sleep disorders, and subjective sleep trouble-we explore the relationship between OBS and these sleep parameters, as well as the potential mediating effects of oxidative stress and inflammation. This research will provide a new theoretical basis for improving sleep quality and offer significant guidance for clinical practice and public health interventions.

## 2 Materials and methods

### 2.1 Study population

**Figure 1:**
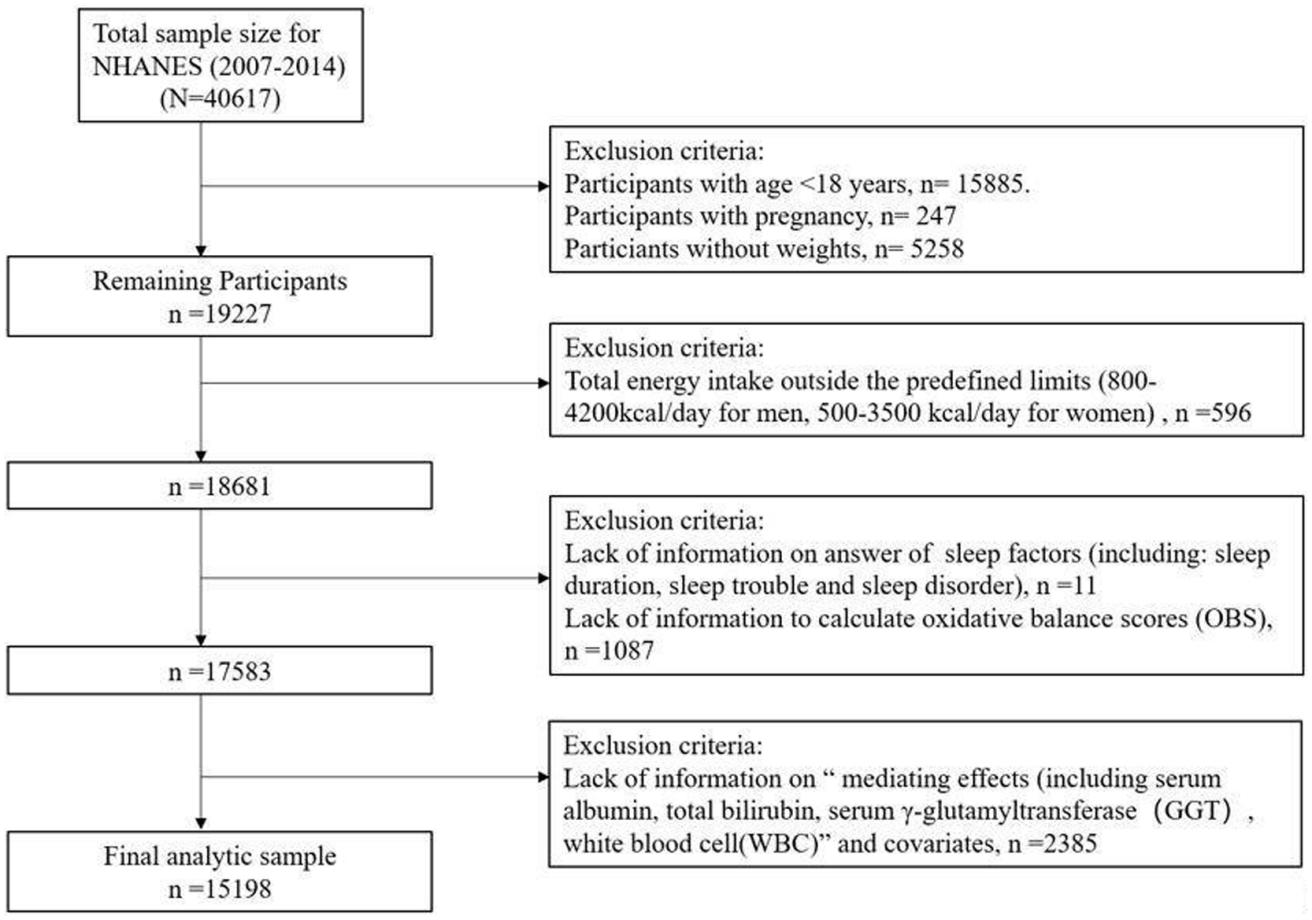
Flow chart of the study population.

This study used data from the NHANES, a nationally representative assessment of the health and nutritional status of the United States population, which is conducted biennially by the National Center for Health Statistics. The survey collects information through a combination of interviews, physical exams, and laboratory analyses, and the data is made publicly available (33). Our study utilized data from four consecutive NHANES survey cycles (2007–2014), as data on lifestyle factors and sleep disorder were not collected before 2007 and after 2014. Among the 40,617 participants, exclusions were made based on the following criteria: (1) age < 18 years (*n* = 15,885), pregnant individuals (*n* = 247), missing weight data (*n* = 5,258); (2) abnormal energy intake (men <800 or > 4,200 kcal/day, women <500 or > 3,500 kcal/day) (*n* = 596); (3) missing sleep (*n* = 11) or OBS (*n* = 1,087) data; and (4) missing information on biomarkers, including albumin, GGT, total bilirubin, and WBC, or covariates (*n* = 2,385). Ultimately, 15,198 participants were included in our analysis (Figure 1).

### 2.2 Oxidative balance scores

In accordance with methodologies outlined in the pertinent literature (34), we computed the individual OBS of each participant. The evaluation of OBS encompassed 16 dietary nutrients (carotenoids, dietary fiber, riboflavin, niacin, vitamin B6, total folate, vitamin B12, vitamin C, vitamin E, calcium, magnesium, zinc, copper, selenium, total fat, and iron) and 4 lifestyle factors (physical activity, alcohol intake, smoking status, and Body Mass Index [BMI]). Based on prior knowledge and findings from preliminary studies, total fat, iron, alcohol, smoking, and BMI were considered pro-oxidants, whereas the remaining factors were deemed antioxidants.

Dietary nutrient intake data were derived from interviews conducted as part of NHANES. Alcohol consumption was determined by calculating the average daily intake of alcoholic beverages over the previous 12 months. Smoking was quantitatively assessed by measuring cotinine, the primary metabolite of nicotine, in the urine. BMI was calculated by dividing the individual’s weight in kilograms by their height in meters squared. Information on physical activity levels was collected using a standardized physical activity questionnaire, and computed with reference to the relevant literature (35).

We divided all elements into three levels based on a weighted distribution. Antioxidant scores ranged from 0 to 2 and pro-oxidants were assigned inverse scores. Subsequently, we separately computed nutrient OBS and lifestyle OBS. Higher nutrient OBS and lifestyle OBS indicated greater exposure to antioxidants, whereas lower scores indicated greater exposure to pro-oxidants. Detailed information regarding the scoring system is provided in Supplementary Table S1.

### 2.3 Sleep quality

Sleep quality is a multidimensional concept encompassing both subjective and objective measures of sleep. It integrates various aspects such as sleep initiation, maintenance, duration, and overall satisfaction with the sleep experience, including feelings of refreshment upon awakening. The term also covers a range of sleep disturbances, including insomnia, hypersomnia, parasomnia, and circadian rhythm disorders, as well as conditions like restless leg syndrome and narcolepsy (36). Similar to previous studies, this research assessed sleep quality using three dimensions, based on data from the NHANES questionnaire (37). These dimensions include: (1) Sleep duration, defined as the total number of hours slept per day; (2) Sleep trouble, defined by self-reported difficulty falling or staying asleep; (3) Sleep disorders, including insomnia and restless leg syndrome, as diagnosed by healthcare professionals. Participants were queried about whether they had reported sleep issues to healthcare professionals, with “yes” responses indicating the presence of sleep trouble. Additionally, participants were asked if they had been informed of the existence of sleep disorders by healthcare professionals, with “yes” responses indicating the presence of sleep disorders. Sleep duration was measured based on participants’ responses to the question: “How much sleep do you get (hours)?” The use of sleep medications was measured based on participants’ responses to the question: “How often take pills to help you sleep?” The frequency of sleep trouble in the depression (abbreviated as “Depression” in the table) over 2 weeks was measured based on participants’ responses to the question: “How often have you been bothered over the last 2 weeks, by the following problems: trouble falling or staying asleep, or sleeping too much?”

### 2.4 Biomarkers

This study utilized biomarkers including GGT, albumin, total bilirubin, and WBC. Biomarker data were obtained through the analysis of peripheral blood samples. Detailed descriptions of the laboratory testing methods used for these biomarkers are available on the NHANES online platform (33).

### 2.5 Covariates

In our study, variables such as sex, age, race/ethnicity, educational level, household income, marital status, depression, the use of sleep medications, and comorbidities were investigated as covariates. Additionally, factors such as caffeine intake and energy intake were considered. Household income was categorized based on the family poverty income ratio (PIR). Comorbidities included diabetes, hyperlipidemia, and cardiovascular diseases.

### 2.6 Statistical analysis

All analyses took into account the sample weights derived from the intricate sampling framework of NHANES. The baseline characteristics of the participants are expressed as mean values and standard deviations (SD) for continuous data, while categorical data are presented as counts(*n*) and proportions(%). The OBS was modeled in tertiles, with the first tertile serving as the reference group. Multivariable logistic regression models were utilized to compute odds ratios (ORs) and 95% confidence intervals(CIs) to evaluate the association between OBS and sleep problems, in addition to sleep disorders. Trends were assessed by treating the median value of the respective intake category as a continuous variable. Weighted linear regression analysis was utilized to compute *β* values along with their respective 95% CIs, delineating the association between OBS and sleep duration. We established three distinct models: Model 1, which underwent adjustments for age and sex; Model 2, which underwent further adjustments for race/ethnicity, marital status, and educational level; Model 3, which underwent additional adjustments for caffeine intake (included because of the antioxidant properties of caffeine), energy intake, and comorbidities; and Model 4 was adjusted by adding variables for depression and the use of sleep medications to Model 3. Furthermore, analyses were stratified by age (<50 years or ≥ 50 years) and sex. Associations linking dietary OBS and lifestyle OBS with sleep quality were separately examined. Additionally, multiple linear regression analyses were performed to assess the relationships between OBS and sleep quality, oxidative stress, and inflammation. Two mediation pathways were constructed to explore the possible mediation mechanisms of oxidative stress and inflammatory markers in linking OBS and sleep quality. The mediation effects of biomarkers were assessed using R software version 4.1.3 (R Foundation for Statistical Computing, Vienna, Austria), adjusting for the covariates used in Model 3. Our results provided the magnitude of indirect effects (β_indirect_), direct effects (β_direct_), total effects (β_total_), proportion mediated (PM), and *p*-values.

Sensitivity analyses were conducted to adjust for the potential impact of depression and sleep medication on the results.

All statistical analyses were conducted using SAS 9.4 (SAS Institute, Cary, NC, United States) and R 4.1.3 (R Foundation, Vienna, Austria), with differences considered statistically significant at a two-sided *p*-value <0.05.

## 3 Results

### 3.1 Baseline participant characteristics

Among the 15,198 NHANES participants analyzed, the average age was 47.3 years; 7,241 (47.53%) were male and 7,957 (52.47%) were female. Those in the highest OBS tertile were more likely to be Non-Hispanic White, married, and have comorbidities than those in the lowest tertile. Moreover, individuals occupying the highest OBS tertile exhibited lower levels of educational attainment, but higher levels of PIR, total energy intake, and caffeine consumption. Notably, participants in the top OBS tertile displayed elevated levels of albumin and total bilirubin, and decreased levels of GGT and WBC compared with their counterparts in the lowest tertile. Importantly, individuals with a higher OBS were less likely to experience sleep issues than those with a lower OBS. Among participants with depression, higher OBS was associated with fewer sleep trouble in 2 weeks. Baseline participant characteristic, categorized into tertiles based on OBS, are presented in Table 1. Additionally, we have provided a breakdown of baseline characteristics by sex in Supplementary Tables S2, S3. In general, the results were consistent between the sexes.

**Table 1:** The baseline characteristics by tertiles of the OBS: National Health and Nutrition Examination Survey 2007–2014 (NHANES 2007–2014).^a^

Characteristics	Total (15,198)	Tertile 1	Tertile 2	Tertile 3	*p* value
Age (years)	47.31 (0.33)	47.64 (0.35)	47.49 (0.42)	46.90 (0.50)	0.1212
PIR	3.00 (0.05)	2.56 (0.06)	3.03 (0.06)	3.35 (0.06)	<0.0001
Energy intake (kcal/day)	2050.95 (9.33)	1586.26 (12.01)	2021.63 (13.27)	2448.00 (14.38)	<0.0001
Caffeine (mg)	167.00 (3.84)	157.08 (4.93)	170.83 (5.34)	171.78 (5.38)	0.0193
Marital status, married (*n*, %)	9,183 (63.44)	2,985 (57.89)	2,879 (64.19)	3,319 (67.28)	<0.0001
Educational level (*n*, %)					<0.0001
College or above	3,527 (15.72)	1,657 (22.70)	1,071 (15.68)	799 (10.16)	
High school or equivalent	3,421 (21.81)	1,392 (26.53)	1,057 (23.19)	972 (16.88)	
Less than high school	8,250 (62.47)	2,297 (50.77)	2,576 (62.13)	3,377 (72.96)	
Race (*n*, %)					<0.0001
Non-Hispanic White	7,293 (69.68)	2,344 (64.06)	2,268 (69.46)	2,681 (74.38)	
Non-Hispanic Black	2,987 (10.35)	1,436 (16.13)	880 (9.84)	671 (6.14)	
Mexican American	2,137 (8.21)	688 (8.05)	688 (8.85)	761 (7.80)	
Others	2,781 (11.76)	878 (11.76)	868 (11.85)	1,035 (11.67)	
Sleep trouble, no (*n*, %)	3,919 (26.68)	1,492 (29.63)	1,210 (26.08)	1,217 (24.79)	0.0019
Sleep disorder, no (*n*, %)	1,339 (8.91)	524 (10.27)	448 (9.42)	367 (7.40)	0.0042
Sleep duration, hours	6.94 (0.02)	6.83 (0.04)	6.94 (0.03)	7.02 (0.03)	0.0006
History of comorbidities, no (*n*, %)	8,330 (60.22)	2,643 (56.49)	2,577 (59.08)	3,110 (64.16)	<0.0001
Biomarkers					
Albumin (g/L)	42.98 (0.06)	42.44 (0.09)	43.02 (0.08)	43.37 (0.08)	<0.0001
GGT (U/L)	27.53 (0.53)	30.44 (1.10)	29.13 (1.15)	23.88 (0.53)	<0.0001
Bilirubin, total (umol/L)	12.44 (0.10)	12.09 (0.15)	12.37 (0.13)	12.78 (0.17)	0.0006
WBC (nmol/L)	7.22 (0.04)	7.54 (0.05)	7.25 (0.05)	6.94 (0.04)	<0.0001
Depression (*n*, %)					<0.0001
0	8,890(58.53)	3,043(16.38)	2,760(18.24)	3,087(22.75)	
≤1 week	3,183(20.95)	1,025(6.74)	979(6.88)	1,179(9.58)	
1 ~ 2 weeks	987(6.50)	407(2.53)	294(2.15)	286(2.05)	
=2 weeks	1,324(8.72)	592(3.46)	405(2.59)	327(1.95)	
Unknown	806(5.30)	274(1.38)	268(1.66)	264(1.66)	
Use of sleep medications (*n*, %)					0.0502
No	3,085(19.23)	1,191(6.67)	921(5.65)	946(6.92)	
Yes	719(4.85)	274(1.60)	223(1.60)	222(1.64)	
Unknown	11,413(75.92)	3,876(22.22)	3,562(24.27)	3,975(29.43)	

a All estimates accounted for complex survey designs in NHANES. Values were mean ± standard error for continuous variables and numbers (percentages) for categorical variables.

OBS, oxidative balance score; PIR, family income-to-poverty ratio; GGT, γ-glutamyl transferase; WBC, white blood cell.

### 3.2 Link between OBS and sleep

Table 2 displays the outcomes derived from weighted logistic regression and linear models (Models 1, 2, and 3). In Model 3, the most extensively adjusted model, an inverse correlation emerged between OBS and both sleep disorders and sleep trouble, with ORs of 0.72 (95% CI, 0.58–0.91; P_trend_ = 0.0055) and 0.81 (95% CI, 0.69–0.96; P_trend_ = 0.0174), for the highest versus the lowest tertile, respectively. Moreover, a higher OBS correlated with extended sleep duration (*β*, 0.009; 95% CI, 0.002–0.016). We further subdivided the OBS into dietary and lifestyle components. Our findings indicated that sleep disorders and sleep trouble were solely influenced by lifestyle OBS in all three models, with ORs of 0.47 (0.38–0.59; P_trend_ < 0.0001) and 0.71 (0.63–0.81; P_trend_ < 0.0001) in Model 3, for the highest vs. lowest tertile, respectively (Table 3). Dietary OBS was significantly associated with sleep duration only (Model 3: *β*, 0.009; 95% CI, 0.002–0.017). The results of the stratified analyses revealed no statistically significant interaction between the OBS and sleep when stratified by sex (*p* > 0.05). When stratified by age (<50 or ≥ 50 years), a statistically significant interaction was found between OBS and sleep trouble (*p* = 0.0118) (Supplementary Table S4). The association was only present in the younger age group (OR, 0.74; 95% CI, 0.59–0.92, for the highest vs. lowest tertile) in Model 3.

**Table 2:** The relationship between OBS and sleep quality in National Health and Nutrition Examination Survey 2007–2014 (NHANES 2007–2014).^a^

Tertile of OBS	Model 1^b^	Model 2^c^	Model 3^d^
Sleep disorder (OR 95%CI)			
Tertile 1	1.00 (reference)	1.00 (reference)	1.00 (reference)
Tertile 2	0.91 (0.72–1.16)	0.92 (0.73–1.16)	0.90 (0.72–1.13)
Tertile 3	0.71 (0.58–0.87)	0.71 (0.58–0.88)	0.72 (0.58–0.91)
*P* trend	0.0015	0.0018	0.0055
Sleep trouble, OR (95%CI)			
Tertile 1	1.00 (reference)	1.00 (reference)	1.00 (reference)
Tertile 2	0.84 (0.73–0.97)	0.86 (0.74–0.99)	0.85 (0.73–0.98)
Tertile 3	0.79 (0.68–0.92)	0.80 (0.69–0.93)	0.81 (0.69–0.96)
*P* trend	0.003	0.0038	0.0174
Sleep duration, *β* estimates (95 CI%)	0.008 (0.002–0.015)	0.010 (0.005–0.016)	0.009 (0.002–0.016)
*p* value	0.0163	0.0004	0.0150

a All estimates accounted for complex survey designs in NHANES.

b Model 1: adjusted for sex and age.

c Model 2: adjusted for age, sex, PIR, marital status and educational level.

d Model 3: adjusted for all variables in model 2 and further for caffeine intake, energy intake and history of comorbidities.

OBS, oxidative balance score; PIR, family income-to-poverty ratio; OR, odds ratio; CI, confidence interval.

**Table 3:** Association between dietary/lifestyle OBS with sleep factors in US adult population, NHANES 2007–2014.^a^

	Model 1^b^	Model 2^c^	Model 3^d^
Dietary OBS			
Sleep disorder (OR 95% CI)			
Tertile 1	1.00 (reference)	1.00 (reference)	1.00 (reference)
Tertile 2	0.95 (0.73–1.22)	0.96 (0.75–1.24)	0.97 (0.75–1.25)
Tertile 3	0.82 (0.65–1.04)	0.83 (0.65–1.05)	0.86 (0.65–1.13)
*P* trend	0.0931	0.1102	0.2707
Sleep trouble, OR (95% CI)			
Tertile 1	1.00 (reference)	1.00 (reference)	1.00 (reference)
Tertile 2	0.88 (0.76–1.02)	0.90 (0.77–1.04)	0.91 (0.77–1.06)
Tertile 3	0.86 (0.74–1.00)	0.87 (0.75–1.01)	0.89 (0.74–1.07)
*P* trend	0.0534	0.0678	0.2018
Sleep duration, *β* estimates (95% CI)	0.01 (0.005–0.017)	0.01 (0.005–0.016)	0.009 (0.002–0.017)
*P* value	0.0004	0.0007	0.0157
Lifestyle OBS			
Sleep disorder (OR 95% CI)			
Tertile 1	1.00 (reference)	1.00 (reference)	1.00 (reference)
Tertile 2	0.66 (0.54–0.80)	0.66 (0.54–0.81)	0.70 (0.57–0.86)
Tertile 3	0.41 (0.33–0.51)	0.41 (0.33–0.52)	0.47 (0.38–0.59)
*P* trend	<0.0001	<0.0001	<0.0001
Sleep trouble, OR (95% CI)			
Tertile 1	1.00 (reference)	1.00 (reference)	1.00 (reference)
Tertile 2	0.81 (0.70–0.93)	0.83 (0.72–0.96)	0.86 (0.74–1.01)
Tertile 3	0.64 (0.56–0.72)	0.65 (0.57–0.73)	0.71 (0.63–0.81)
*P* trend	<0.0001	<0.0001	<0.0001
Sleep duration, *β* estimates (95% CI)	0.02 (−0.004–0.045)	0.02 (−0.003–0.029)	0.009 (−0.013–0.031)
*p* value	0.122	0.0987	0.4106

a All estimates accounted for complex survey designs in NHANES.

b Model 1: adjusted for sex and age.

c Model 2: adjusted for age, sex, family income-to-poverty ratio, marital status and educational level.

d Model 3: adjusted for all variables in model 1 and further for caffeine intake, energy intake and history of comorbidities.

OBS, oxidative balance score; OR, odds ratio; CI, confidence interval.

### 3.3 Mediation effects of individual and multiple factors

**Figure 2:**
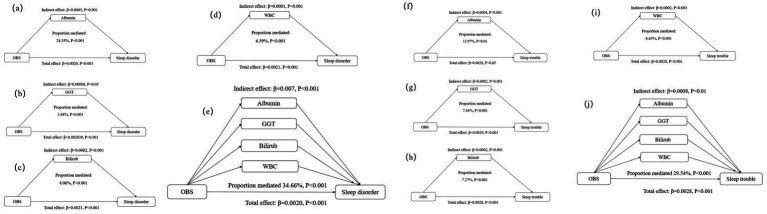
Estimated proportion of the association between OBS and sleep disorder mediated by oxidative stress and inflammatory markers: (a) Albumin; (b) GGT; (c) Bilirubin; (d) WBC; (e) Albumin, GGT, Bilirubin, and WBC. Estimated proportion of the association between OBS and sleep trouble mediated by oxidative stress and inflammatory markers: (f) Albumin; (g) GGT; (h) Bilirubin; (i) WBC; (j) Albumin, GGT, Bilirubin, and WBC. All analyses were adjusted for age, sex, family income-to-poverty ratio, marital status and educational level, caffeine intake, energy intake and history of comorbidities. OBS, oxidative balance score; GGT, y-glutamyl transferase; WBC, white blood cell.

Compared with participants with a higher OBS, those with a lower OBS had higher levels of GGT and WBC counts, but lower albumin and total bilirubin levels (Supplementary Table S5). The observed association of the OBS with sleep disorders and sleep trouble was mediated by albumin, GGT, total bilirubin, and WBC. Specifically, separate mediation analyses revealed that albumin mediated 24.55 and 12.97% of the total relationship between the OBS and sleep disorders and sleep trouble, respectively. GGT accounted for 3.68 and 7.44%, respectively; total bilirubin mediated 8.06 and 7.27%, respectively; and WBC mediated 6.59 and 6.63%, respectively (Table 4; Figure 2). Multiple mediation analysis showed that all the potential biomarkers (albumin, GGT, bilirubin, and WBC) combined mediated 34.66 and 29.54% of the total relationship for sleep disorders and sleep trouble, respectively (Table 4; Figure 2). No significant results were found in the mediation analysis of OBS and sleep duration (Table 4; Figure 2).

**Table 4:** Mediation analyses with separate and multiple mediators between OBS and sleep factors (*n* = 15,198) in National Health and Nutrition Examination Survey 2007–2014 (NHANES 2007–2014).^a^

	Indirect effect	Direct effect	Total effect	Prop. Mediated
	*β*_indirect_ (95% CI)	*β*_direct_ (95% CI)	*β*_total_ (95% CI)	%
Sleep disorder				
Separate mediators				
Albumin	0.0005 (0.0004–0.0006)***	0.0016 (0.0008–0.0024)***	0.0020 (0.0013–0.0029)***	24.55***
GGT	0.00008 (0.00003–0.00015)*	0.0020 (0.0014–0.0026)***	0.002039 (0.0014–0.0027)***	3.68***
Bilirubin	0.0002 (0.0001–0.0003)***	0.0019 (0.0013–0.0027)***	0.0021 (0.0015–0.0028)***	8.06***
WBC	0.0001 (0.00006–0.0002)***	0.0020 (0.0012–0.0028)***	0.0021 (0.0014–0.0029)***	6.59***
Multiple mediators (including Albumin, GGT, bilirubin and WBC)	0.0007 (0.0005–0.0009)***	0.0013 (0.0005–0.0021)***	0.0020 (0.0012–0.0027)***	34.66***
Sleep trouble				
Separate mediators				
Albumin	0.0004 (0.0002–0.0005)***	0.0024 (0.0010–0.0038)***	0.0028 (0.0015–0.0003)*	12.97**
GGT	0.0002 (0.0001–0.0003)***	0.0027 (0.0016–0.0039)***	0.0029 (0.0018–0.0040)***	7.44***
Bilirubin	0.0002 (0.0001–0.0003)***	0.0026 (0.0015–0.0037)***	0.0028 (0.0017–0.0039)***	7.27***
WBC	0.0002 (0.0001–0.0003)***	0.0026 (0.0014–0.0037)***	0.0028 (0.0017–0.0038)***	6.63***
Multiple mediators (including Albumin, GGT, bilirubin and WBC)	0.0008 (0.0006–0.0010)**	0.0019 (0.0007–0.0031)***	0.0028 (0.0016–0.0040)***	29.54***
Sleep duration				
Separate mediators				
Albumin	0.0014 (0.0003–0.0023)**	0.0066 (−0.0008–0.0151)	0.0080 (0.0012–0.0164)*	16.62
GGT	−0.0005 (−0.0021–0.0007)	0.0086 (0.0012–0.0170)*	0.0081 (0.00002–0.0168)	NA
Bilirubin	0.00006 (−0.00030–0.00046)	0.0081 (0.0016–0.0159)***	0.0082 (0.0020–0.0160)*	0.63
WBC	−0.0003 (−0.0013–0.00050)	0.0081 (0.0003–0.0169)*	0.0077 (0.0003–0.0162)	NA

a All estimates accounted for complex survey designs in NHANES. All analyses were adjusted for age, sex, family income-to-poverty ratio, marital status and educational level, caffeine intake, energy intake and history of comorbidities.

OBS, oxidative balance score; GGT, γ-glutamyl transferase; WBC, white blood cell. *p < 0.05; **p < 0.01; ***p < 0.001.

Sensitivity analyses revealed a significant association between OBS and sleep disorder. The OR for OBS was 0.78 (95% CI: 0.61–0.99, *p* = 0.0421) (Supplementary Table S6).

## 4 Discussion

Our findings suggest that an elevated OBS correlates with enhanced sleep quality, marked by decreased sleep disorders, mitigated sleep trouble, and prolonged sleep duration. Notably, oxidative stress and inflammatory factors partially mediate the relationship between OBS and sleep disorders.

This study utilized demographic data from the NHANES database, a cross-sectional survey representing the entire population of the United States, to explore the correlation between OBS and sleep quality, as well as the potential mechanisms underlying this association. The central focus of our analysis was the OBS. Our results robustly affirm the notion that an elevated OBS is closely linked to improved sleep quality among American adults. These findings are consistent with prior research suggesting that modifications in dietary elements, such as increasing dietary fiber intake (38), optimizing serum zinc levels and zinc/copper and zinc/selenium ratios (39), and augmenting vitamin C intake (40), significantly enhance sleep quality by elevating the OBS. Likewise, embracing healthier lifestyle habits, such as refraining from smoking (41) and participating in moderate to vigorous physical activity (42), has been demonstrated to increase OBS levels, thereby enhancing sleep quality. Moreover, individuals with an elevated BMI and those classified as overweight or obese are more susceptible to suboptimal sleep quality than individuals with a lower BMI (24). A previous cross-sectional study involving American adults revealed the potential influence of diet and lifestyle factors on sleep quality, through their effects on oxidative stress (43). However, this specific investigation did not investigate the mechanisms underlying this association.

Our study demonstrated an association between the OBS and sleep disorders, sleep trouble, and sleep duration. Specifically, dietary OBS was correlated with sleep duration, whereas lifestyle OBS was associated with sleep disorders and sleep trouble. This suggests that distinct aspects of sleep are influenced by different OBS components. Enhancing sleep quality and quantity therefore requires the consideration of both dietary and lifestyle factors. However, after adjusting for variables such as depression and the use of sleep medications, the association was limited to a reduction in sleep disorders, which is inconsistent with the main results. This discrepancy may be attributed to incomplete data or insufficient expression of certain data aspects.

Although the mechanism by which the OBS affects sleep remains unclear, studies have shown that OBS regulates oxidative stress (44), levels of albumin (45), levels of 25-hydroxyvitamin D (43), and WBC counts. Sleep and inflammation are closely related, and previous studies have demonstrated a positive association between CRP concentrations and both subjectively and objectively assessed poor sleep quality, as well as insufficient sleep duration (46, 47). Chronic sleep deprivation has been associated with low-grade inflammation (48). Experimental sleep deprivation leads to elevated levels of circulating inflammatory markers (49, 50). Additionally, *in vitro* studies have provided evidence that quetiapine significantly diminishes the release of proinflammatory cytokines from microglial cells, into the cerebrospinal fluid, leading to improved sleep quality (51).

The multiple mediation analysis conducted in our study demonstrated that oxidative stress factors, including albumin, total bilirubin, and GGT, along with WBC, a marker of inflammation, mediated the association between OBS and sleep quality. These results support the involvement of oxidative stress and inflammation in the relationship between OBS and sleep quality.

Modulating the OBS can impact sleep quality through oxidative stress and inflammatory factors. Previous studies and our own findings suggest various potential mechanisms: (1) Antioxidant effects. Increased antioxidant capacity leads to the scavenging of free radicals and therefore, enhanced sleep quality (52). Previous studies have suggested that antioxidant administration may ameliorate sleep disorders and regulate sleep cycles (53). (2) Anti-inflammatory effects. Inflammatory mediators can traverse the blood–brain barrier, thereby affecting neurons and neurotransmitters in the brain, and disturbing sleep patterns (54, 55). (3) Neurotransmitter balance. Oxidative stress can decrease the levels of neurotransmitters including dopamine and serotonin (56). Interleukin-1β and tumor necrosis factor-*α*, among other inflammatory factors, have the potential to hinder neurotransmitter synthesis and release, resulting in an imbalance that could subsequently impact sleep patterns (57). (4) Maintenance of vascular homeostasis. Oxidative stress may impair the functionality of endothelial cells that line blood vessels, reducing vasodilation and potentially leading to hypertension and other vascular conditions (58). Inflammation can also cause endothelial dysfunction and disrupt vascular performance (59), culminating in inadequate blood flow, thus affecting cerebral perfusion and disturbing sleep (60). (5) Hormonal regulation. Oxidative stress and inflammation can modulate the secretion of hormones, including those pertinent to sleep regulation (61).

This study has several strengths. Firstly, the OBS, a detailed composite score integrating dietary and lifestyle factors, was used to evaluate antioxidant status. Secondly, a large, nationally representative sample was employed to explore the link between OBS and sleep patterns. Thirdly, mediation analyses were conducted to investigate the mechanisms underlying the OBS–sleep relationship. However, this study also has certain limitations. Firstly, the reliance on self-reported sleep data may have introduced a degree of imprecision into the results. Secondly, the estimation of dietary components was contingent upon the averaging of two 24-h recalls, potentially introducing bias. Thirdly, the presence of unaccounted for and unmeasured confounding variables may have influenced the results. Lastly, due to the cross-sectional design of the study, causal relationships could not be established. Cautious interpretation of our research findings is therefore recommended.

## 5 Conclusion

The present study observed a positive correlation between OBS and sleep quality. Moreover, our data indicate that albumin levels, total bilirubin levels, GGT levels, and WBC counts may modulate the connection between OBS and sleep. To substantiate this relationship and clarify its underlying mechanisms, further prospective and experimental investigations are essential.

## Data Availability

The original contributions presented in the study are included in the article/Supplementary material, further inquiries can be directed to the corresponding author.
