# Supplementary material for: Oxidative stress and inflammation mediate the association between elevated oxidative balance scores and improved sleep quality: evidence from NHANES
Source: Front Nutr. 2024 Oct 18;11:1469779. doi: 10.3389/fnut.2024.1469779 (PMC11528468; doi:10.3389/fnut.2024.1469779)
Supplement: Supplementary file 1 [file Table_1.docx]

Supplementary Table 1: Oxidative balance score assignment scheme.

| OBS components | Property | Male | | | Female | | |
| --- | --- | --- | --- | --- | --- | --- | --- |
| **Dietary OBS components** |  | 0 | 1 | 2 | 0 | 1 | 2 |
| Dietary fiber (g/d) | A | <13.15 | 13.15-20.7 | ≥20.7 | <11.25 | 11.25-16.95 | ≥16.95 |
| Carotene (RE/d) | A | <667.50 | 667.50-2326.00 | ≥2326.00 | <686.50 | 686.50-2499.00 | ≥2499.00 |
| Riboflavin (mg/d) | A | <1.74 | 1.74-2.51 | ≥2.51 | <1.38 | 1.38-1.96 | ≥1.96 |
| Niacin (mg/d) | A | <22.28 | 22.28-31.66 | ≥31.66 | <16.15 | 16.15-22.87 | ≥22.87 |
| Vitamin B6 (mg/d) | A | <1.73 | 1.73-2.53 | ≥2.53 | <1.30 | 1.30-1.88 | ≥1.88 |
| Total folate (mcg/d) | A | <328.50 | 328.50-490.00 | ≥490.00 | <261.00 | 261.00-383.00 | ≥383.00 |
| Vitamin B12 (mcg/d) | A | <3.64 | 3.64-6.14 | ≥6.14 | <2.65 | 2.65-4.53 | ≥4.53 |
| Vitamin C (mg/d) | A | <42.65 | 42.65-101.25 | ≥101.25 | <40.4 | 40.4-90.45 | ≥90.45 |
| Vitamin E (ATE) (mg/d) | A | <5.88 | 5.88-9.14 | ≥9.14 | <4.78 | 4.78-7.6 | ≥7.6 |
| Calcium (mg/d) | A | <736.00 | 736.00-1110.00 | ≥1110.00 | <618.00 | 618.00-920.00 | ≥920.00 |
| Magnesium (mg/d) | A | <255.50 | 255.50-354.50 | ≥354.50 | <206.00 | 206.00-283.50 | ≥283.50 |
| Zinc (mg/d) | A | <9.71 | 9.71-14.06 | ≥14.06 | <7.23 | 7.23-10.29 | ≥10.29 |
| Copper (mg/d) | A | <1.05 | 1.05-1.47 | ≥1.47 | <0.86 | 0.86-1.20 | ≥1.20 |
| Selenium (mcg/d) | A | <101.00 | 101.00-140.60 | ≥140.60 | <73.45 | 73.45-103.85 | ≥103.85 |
| Total fat (g/d) | P | <66.64 | 66.64-96.89 | ≥96.89 | <49.65 | 49.65-73.13 | ≥73.13 |
| Iron (mg/d) | P | <12.58 | 12.58-18.17 | ≥18.17 | <9.81 | 9.81-14.02 | ≥14.02 |
| **Lifestyle OBS components** |  |  |  |  |  |  |  |
| Physical activity (MET-minute/week) | A | <620.00 | 620.00-3600.00 | ≥3600.00 | <180.00 | 180.00-1640.00 | ≥1640.00 |
| Alcohol (g/d) | P | ≥30.00 | 0-30.00 | None | ≥15.00 | 0.00-15.00 | None |
| Body mass index (kg/m^2^) | P | <25.91 | 25.91-30.20 | ≥30.20 | <25.44 | 25.44-31.51 | ≥31.51 |
| Cotinine (ng/mL) | P | <0.02 | 0.02-0.68 | ≥0.68 | <0.02 | 0.02-0.10 | ≥0.10 |

A stood for the antioxidant, P for the pro-oxidant, RE for the retinal equivalent, ATE for the alpha-tocopherol equivalent, and MET for the metabolic equivalent.

Supplementary Table 2: The baseline characteristics of males by tertiles of the OBS: National Health and Nutrition Examination Survey 2007–2014 (NHANES 2007-2014)^1^.

| Characteristics | Total (7241) | Tertile 1 | Tertile 2 | Tertile 3 | *P* value |
| --- | --- | --- | --- | --- | --- |
| Age (years) | 46.75 (0.37) | 47.71 (0.42) | 47.16 (0.60) | 45.64 (0.55) | 0.003 |
| PIR | 3.13 (0.05) | 2.73 (0.07) | 3.18 (0.06) | 3.41 (0.07) | <.0001 |
| Energy intake (kcal/day) | 2369.55 (13.49) | 1828.73(17.61) | 2317.29 (21.01) | 2840.09 (18.13) | <.0001 |
| Caffeine (mg) | 186.94 (5.06) | 178.27 (6.95) | 188.52 (8.55) | 192.44 (7.87) | 0.06 |
| Marital status, Married (n, %) | 4856 (66.93) | 1593 (62.01) | 1545 (67.79) | 1718 (70.10) | 0.0014 |
| Educational level (n, %) |  |  |  |  | <.0001 |
| College or above | 1728 (15.71) | 803 (23.07) | 513 (14.78) | 412 (10.68) |  |
| High school or equivalent | 1697 (22.28) | 686 (26.08) | 519 (23.05) | 492 (18.64) |  |
| Less than high school | 3816 (62.01) | 1062 (50.84) | 1218 (62.17) | 1536 (70.68) |  |
| Race (n, %) |  |  |  |  | <.0001 |
| Non-Hispanic White | 3539 (69.78) | 1153 (65.12) | 1133 (70.76) | 1253 (72.63) |  |
| Non-Hispanic Black | 1373 (9.17) | 670(14.22) | 385 (8.41) | 318 (5.83) |  |
| Mexican American | 1039 (9.20) | 300 (8.52) | 347 (9.78) | 392 (9.24) |  |
| Others | 1290 (11.85) | 428 (12.14) | 385 (11.05) | 477 (12.30) |  |
| Sleep trouble, yes (n, %) | 1576 (22.84) | 591 (24.78) | 492(21.58) | 493 (22.38) | 0.1085 |
| Sleep disorder, yes (n, %) | 693 (9.74) | 272 (11.25) | 233 (9.67) | 188 (8.62) | 0.2412 |
| Sleep duration, hours | 6.88 (0.03) | 6.75 (0.05) | 6.95 (0.05) | 6.93 (0.04) | 0.01 |
| History of comorbidities, no (n, %) | 3952 (60.12) | 1249(56.41) | 1233(59.49) | 1470 (63.57) | 0.006 |
| Biomarkers |  |  |  |  |  |
| Albumin (g/L) | 43.95 (0.07) | 43.42 (0.11) | 44.02 (0.10) | 44.31 (0.10) | <.0001 |
| GGT (U/L) | 31.51 (0.59) | 34.36 (0.88) | 33.12 (1.44) | 27.90 (0.74) | <.0001 |
| Bilirubin, total (umol/L) | 13.86 (0.12) | 13.60 (0.19) | 13.63 (0.19) | 14.25 (0.22) | 0.006 |
| WBC (nmol/L) | 7.20 (0.05) | 7.60 (0.07) | 7.16 (0.07) | 6.92 (0.07 ) | <.0001 |
| Depression (n, %) |  |  |  |  | <.0001 |
| 0 | 4630(63.99) | 1618(17.69) | 1445(20.42) | 1567(24.13) |  |
| ≤1 week | 1376(19.02) | 417(5.95) | 429(6.40) | 530(9.28) |  |
| 1~2weeks | 423(5.85) | 185(2.28) | 126(1.96) | 112(1.66) |  |
| =2weeks | 511(7.06) | 228(2.90) | 153(2.07) | 130(1.63) |  |
| Unknown | 295(4.08) | 99(1.09) | 97(1.25) | 99(1.30) |  |
| Use of sleep medications (n, %) |  |  |  |  | 0.1349 |
| No | 1521(20.00) | 582(6.83) | 464(5.89) | 475(7.28) |  |
| Yes | 288(3.43) | 101(0.97) | 95(1.29) | 92(1.17) |  |
| Unknown | 5426(76.56) | 1864(22.11) | 1691(24.91) | 1871(29.55) |  |

^1^ All estimates accounted for complex survey designs in NHANES. Values were mean ± standard error for continuous variables and numbers (percentages) for categorical variables. Abbreviation and acronyms: OBS, oxidative balance score; PIR family income-to-poverty ratio; GGT γ-glutamyl transferase; WBC white blood cell.

Supplementary Table 3: The baseline characteristics of females by tertiles of the OBS: National Health and Nutrition Examination Survey, United States, 2007–2014 (NHANES 2007-2014)^1^.

| Characteristics | Total (7957) | Tertile 1 | Tertile 2 | Tertile 3 | *P* value |
| --- | --- | --- | --- | --- | --- |
| Age (years) | 47.83 (0.37) | 47.59 (0.45) | 47.81 (0.46) | 48.05 (0.58) | 0.88 |
| PIR | 2.89 (0.05) | 2.40 (0.06) | 2.88 (0.06) | 3.29 (0.06) | <.0001 |
| Energy intake (kcal/day) | 1762.00 (9.14) | 1373.87 (12.89) | 1744.40 (12.52) | 2093.26 (15.54) | <.0001 |
| Caffeine (mg) | 148.93 (3.60) | 138.51 (5.08) | 154.25 (5.69) | 153.09 (5.49) | 0.11 |
| Marital status, Married (n, %) | 4327 (60.28) | 1392 (16.82) | 1334 (18.82) | 1601 (24.63) | <.0001 |
| Educational level (n, %) |  |  |  |  | <.0001 |
| College or above | 1799 (15,74) | 854 (6.93) | 558 (5.11) | 387 (3.69) |  |
| High school or equivalent | 1724 (21.38) | 706 (8.34) | 538 (7.22) | 480 (5.82) |  |
| Less than high school | 4434 (62.88) | 1235 (15.72) | 1358 (18.61) | 1841 (28.55) |  |
| Race (n, %) |  |  |  |  | <.0001 |
| Non-Hispanic White | 3754 (69.59) | 1191 (19.57) | 1135 (21.11) | 1428 (28.91) |  |
| Non-Hispanic Black | 1614 (11.42) | 766 (5.52) | 495 (3.46) | 353 (2.44) |  |
| Mexican American | 1098 (7.31) | 388 (2.37) | 341 (2.47) | 369 (2.48) |  |
| Others | 1491 (11.67) | 450 (3.54) | 483 (3.90) | 558 (4.23) |  |
| Sleep trouble, no (n, %) | 2343 (0.92) | 901 (0.61) | 718 (0.48) | 724 (0.55) | 0.0012 |
| Sleep disorder, no (n, %) | 646 (0.47) | 252 (0.32) | 215 (0.26) | 179 (0.47) | 0.0093 |
| Sleep duration, hours | 6.98 (0.03) | 6.90 (0.06) | 6.93 (0.05) | 7.10 (0.03) | 0.03 |
| History of comorbidities, no (n, %) | 4378 (0.98) | 1394 (0.69) | 1344 (0.58) | 1640 (1.09) | 0.0004 |
| Biomarkers |  |  |  |  |  |
| Albumin (g/L) | 42.09 (0.07) | 41.59 (0.10) | 42.09 (0.10) | 42.52 (0.09) | <.0001 |
| GGT (U/L) | 23.93 (0.81) | 27.00 (1.92) | 25.39 (1.74) | 20.23 (0.62) | <.0001 |
| Bilirubin, total (umol/L) | 11.15 (0.10) | 10.76 (0.15) | 11.18 (0.14) | 11.45 (0.16) | 0.0007 |
| WBC (nmol/L) | 7.24 (0.05) | 7.48 (0.07) | 7.33 (0.07) | 6.96 (0.06) | <.0001 |
| Depression (n, %) |  |  |  |  | <.0001 |
| 0 | 4630(63.99) | 1618(17.69) | 1445(20.42) | 1567(24.13) |  |
| ≤1 week | 1376(19.02) | 417(5.95) | 429(6.40) | 530(9.28) |  |
| 1~2weeks | 423(5.85) | 185(2.28) | 126(1.96) | 112(1.66) |  |
| =2weeks | 511(7.06) | 228(2.90) | 153(2.07) | 130(1.63) |  |
| Unknown | 295(4.08) | 99(1.09) | 97(1.25) | 99(1.30) |  |
| Use of sleep medications (n, %) |  |  |  |  | 0.1349 |
| No | 1521(20.00) | 582(6.83) | 464(5.89) | 475(7.28) |  |
| Yes | 288(3.43) | 101(0.97) | 95(1.29) | 92(1.17) |  |
| Unknown | 5426(76.56) | 1864(22.11) | 1691(24.91) | 1871(29.55) |  |

^1^ All estimates accounted for complex survey designs in NHANES. Values were mean ± standard error for continuous variables and numbers (percentages) for categorical variables. Abbreviation and acronyms: family income-to-poverty ratio family income-poverty ratio. Abbreviation and acronyms: OR Odd ratio; GGT γ-glutamyl transferase; WBC white blood cell.

Supplementary Table 4: Stratified analysis of associations of OBS with sleep factors in US adult population, NHANES 2007–2014 ^1^.

| Stratified factors | Estimate |  | *P* for interaction |
| --- | --- | --- | --- |
| **Sex** |  |  |  |
|  | Sleep disorder (OR 95%CI) |  | 0.5394 |
| Male | Tertile 1 | 1.00 (reference) |  |
|  | Tertile 2 | 0.82(0.60-1.01) |  |
|  | Tertile 3 | 0.76(0.53-1.07) |  |
| Female | Tertile 1 | 1.00 (reference) |  |
|  | Tertile 2 | 1.00(0.74-1.35) |  |
|  | Tertile 3 | 0.70(0.50-0.98) |  |
|  | Sleep trouble, OR (95%CI) |  | 0.0885 |
| Male | Tertile 1 | 1.00 (reference) |  |
|  | Tertile 2 | 0.86(0.72-1.03) |  |
|  | Tertile 3 | 0.75(0.61-0.93) |  |
| Female | Tertile 1 | 1.00 (reference) |  |
|  | Tertile 2 | 0.83(0.63-1.08) |  |
|  | Tertile 3 | 0.91(0.69-1.19) |  |
|  | Sleep duration, *β* estimates (95 CI%) |  | 0.3273 |
| Male | Continuous | 0.013(0.004-0.023) |  |
| Female | Continuous | 0.004(-0.007-0.015) |  |
| **Age (years)** |  |  |  |
|  | Sleep disorder (OR 95%CI) |  | 0.3005 |
| Age < 50 | Tertile 1 | 1.00 (reference) |  |
|  | Tertile 2 | 0.86(0.60-1.22) |  |
|  | Tertile 3 | 0.74(0.51-1.08) |  |
| Age ≥ 50 | Tertile 1 | 1.00 (reference) |  |
|  | Tertile 2 | 0.95(0.69-1.30) |  |
|  | Tertile 3 | 0.73(0.54-0.98) |  |
|  | Sleep trouble, OR (95%CI) |  | 0.0118 |
| Age < 50 | Tertile 1 | 1.00 (reference) |  |
|  | Tertile 2 | 0.73(0.59-0.90) |  |
|  | Tertile 3 | 0.74(0.59-0.92) |  |
| Age ≥ 50 | Tertile 1 | 1.00 (reference) |  |
|  | Tertile 2 | 1.02(0.83-1.26) |  |
|  | Tertile 3 | 0.95(0.75-1.20) |  |
|  | Sleep duration, *β* estimates (95 CI%) |  | 0.3670 |
| Age < 50 | Continuous | 0.011(-0.000-0.023) |  |
| Age ≥ 50 | Continuous | 0.002(-0.005-0.009) |  |

Supplementary Table 5: Associations of OBS with biomarkers in National Health and Nutrition Examination Survey 2007–2014 (NHANES 2007-2014) ^1^.

| Biomarkers | Model 1 ^2^ | Model 2 ^3^ | Model 3 ^4^ |
| --- | --- | --- | --- |
|  | *β* estimates (95 CI%), *P*-value | | |
| **Oxidative stress** |  |  |  |
| Albumin | 0.008 (0.001 - 0.015),  0.0163 | 0.046(0.036-0.056),  <.0001 | 0.075(0.063-0.087),  <.0001 |
| GGT | -0.058 (-0.544--0.311),  <.0001 | -0.387(-0.531--0.243),  <.0001 | 0.520 (-0.653--0.387),  <.0001 |
| Bilirubin | 0.045 (0.020 - 0.070),  0.0005 | 0.028(0.003-0.053),  0.0262 | 0.059(0.030-0.088),  0.0001 |
| **Inflammatory factors** |  |  |  |
| WBC | -0.040 (-0.050 - 0.030),  <.0001 | -0.029(-0.037--0.021),  <.0001 | -0.034 (-0.043--0.025),  <.0001 |

^1^ All estimates accounted for complex survey designs in NHANES. ^2^ Model 1: adjusted for sex and age. ^3^ Model 2: adjusted for age, sex, family income-to-poverty ratio, marital status and educational level. ^4^ Model 3: adjusted for all variables in model 1 and further for caffeine intake, energy intake and history of comorbidities. Abbreviation: OBS, oxidative balance score; GGT, γ-glutamyl transferase; WBC, white blood cell, *β*, beta; CI, confidence interval.

Supplementary Table 6: The relationship between OBS and sleep quality in National Health and Nutrition Examination Survey 2007–2014 (NHANES 2007-2014) ^1^ in Model4^2^.

| Type of sleep quality | OBS | Dietary OBS | Lifestyle OBS |
| --- | --- | --- | --- |
| Sleep disorder (OR 95%CI) |  |  |  |
| Tertile 1 | 1.00 (reference) | 1.00 (reference) | 1.00 (reference) |
| Tertile 2 | 0.93(0.73-1.18) | 1.00(0.77-1.29) | 0.73(0.59-0.90) |
| Tertile 3 | 0.78(0.61-0.99) | 0.92(0.69-1.23) | 0.51(0.41-0.64) |
| *P* trend | 0.0421 | 0.5625 | <.0001 |
| Sleep trouble, OR (95%CI) |  |  |  |
| Tertile 1 | 1.00 (reference) | 1.00 (reference) | 1.00 (reference) |
| Tertile 2 | 0.90(0.76-1.07) | 0.95(0.80-1.14) | 0.92(0.78-1.1) |
| Tertile 3 | 0.93(0.77-1.13) | 0.99(0.81-1.21) | 0.80(0.70-0.92) |
| *P* trend | 0.4639 | 0.93 | 0.0017 |
| Sleep duration, *β* estimates (95 CI%) | 0.0063(-0.0008-0.0134) | 0.0075(-0.0003-0.0153) | -0.0004（-0.0226-0.0217） |
| *P* value | 0.0806 | 0.0586 | 0.97 |

^1^ All estimates accounted for complex survey designs in NHANES. ^2^Model 4: adjusted for age, sex, PIR, marital status, educational level, caffeine intake, energy intake and history of comorbidities, depression and the pills to help sleep. Abbreviation: OBS, oxidative balance score; PIR, family income-to-poverty ratio; OR, odds ratio; CI, confidence interval.
